# PPARγ as a therapeutic target to rescue mitochondrial function in neurological disease

**DOI:** 10.1016/j.freeradbiomed.2016.06.023

**Published:** 2016-11

**Authors:** Juan Carlos Corona, Michael R. Duchen

**Affiliations:** a Department of Cell and Developmental Biology, University College London, London WC1E 6BT, United Kingdom; b Laboratory of Neurosciences, Hospital Infantil de México Federico Gómez, Mexico City, Mexico

**Keywords:** PPARγ, peroxisome proliferation-activated receptor gamma, 15D-PGJ2, 15-deoxy-Δ^12,14^-Prostaglandin J2, TZDs, thiazolidinediones, CNS, central nervous system, WAT, white adipose tissue, BAT, brown adipose tissue, SRC, steroid receptor coactivator, CBP, CREB-binding protein, AF-1, transcriptional activation domain, PGC1α, peroxisome proliferator-activated receptor gamma coactivator 1α, TFAM, mitochondrial transcription factor A, mtDNA, mitochondrial DNA, UCP-1, uncoupling protein 1, UCP-2, uncoupling protein 2, NRF1, nuclear respiratory factor 1, NRF2, nuclear respiratory factor 2, ROS, reactive oxygen species, SOD1, superoxide dismutase 1, TNFα, tumour necrosis factor α, Bcl-2, B-cell lymphoma2, Bax, Bcl-2-associated X protein, MPP^+^, 1-methyl-4-phenylpyridinium ion, iNOS, inducible nitric oxide synthase, COX-2, cyclooxygenase-2, LPS, lipopolysacchacaride, ΔΨ_m_, mitochondrial membrane potential, ALS, amyotrophic lateral sclerosis, HD, huntington's disease, AD, alzheimer's disease, PD, parkinson's disease, MPTP, 1-methyl-4-phenyl-1,2,3,6-tetrahydropyridine, Nrf2, nuclear factor erythroid-derived 2-like 2, NF-κB, nuclear factor-κB, HO-1, haem oxygenase-1, TDP-43, TAR DNA-binding protein 43, BDNF, brain derived neurotrophic factor, A*β*, protein amyloid-*β*, GSK-3*β*, glycogen synthase kinase-3*β*, Cdk5, cyclin-dependent kinase 5, 6-OHDA, 6-hydroxydopamine, PPARγ agonists, Mitochondrial function, Neurodegenerative disorders, Neuroprotection

## Abstract

There is increasing evidence for the involvement of mitochondrial dysfunction and oxidative stress in the pathogenesis of many of the major neurodegenerative and neuroinflammatory diseases, suggesting that mitochondrial and antioxidant pathways may represent potential novel therapeutic targets. Recent years have seen a rapidly growing interest in the use of therapeutic strategies that can limit the defects in, or even to restore, mitochondrial function while reducing free radical generation. The peroxisome proliferation-activated receptor gamma (PPARγ), a ligand-activated transcription factor, has a wide spectrum of biological functions, regulating mitochondrial function, mitochondrial turnover, energy metabolism, antioxidant defence and redox balance, immune responses and fatty acid oxidation. In this review, we explore the evidence for potential beneficial effects of PPARγ agonists in a number of neurological disorders, including Parkinson’s disease, Alzheimer’s disease, Amyotrophic lateral sclerosis and Huntington’s disease, ischaemia, autoimmune encephalomyelitis and neuropathic pain. We discuss the mechanisms underlying those beneficial effects in particular in relation to mitochondrial function, antioxidant defence, cell death and inflammation, and suggest that the PPARγ agonists show significant promise as therapeutic agents in otherwise intractable neurological disease.

## Highlights

• PPARγ promotes oxidative phosphorylation, antioxidant defence and mitochondrial biogenesis.
• A number of pharmacological agents activate PPARγ.
• Mitochondrial dysfunction and inflammation underpin many neurodegenerative diseases.
• PPARγ agonists may prove beneficial for the management of neurodegenerative disease.

## 1 Introduction

The peroxisome proliferator-activated receptor (PPAR) was originally cloned from liver peroxisomes, which are organelles that participate in the metabolism of fatty acids [77]. PPARs are ligand-inducible transcription factors that belong to the hormone nuclear receptor superfamily. PPAR activation is involved in cellular differentiation and proliferation, angiogenesis, vascular protection and regulation of blood pressure. PPAR activation is also anti-apoptotic, anti-oxidant, anti-inflammatory, and limits cancer metastasis [129], [13], [160], [19], [24], [41], [44]. In addition to these effects, PPARs also play key roles in the regulation of metabolism, regulating insulin sensitivity, mitochondrial biogenesis, and glucose and lipid homoeostasis [15], [168], [23]. As lipid sensors, PPARs modulate metabolism in response to dietary lipid intake and direct lipid storage and metabolism [106].

Three members of the PPAR family have been cloned, encoded by three distinct genes located on human chromosomes 22, 6 and 3: PPARα (NR1C1), PPARβ/δ (NR1C2), and PPARγ (NR1C3) respectively [48]. While the three PPAR isoforms share a high degree of structural similarity, they have distinct physiological roles, ligand specificity and tissue distribution. Mutations of each of the PPAR isoforms has been associated with hereditary disease. Some mutations of the PPARs lead to a loss of function, resulting in familial partial lipodystrophy, acanthosis nigricans or insulin resistance [10], [173], [26]. Dysfunction of each of the three PPAR isoforms has been specifically implicated in dysfunction in the central nervous system (CNS), suggesting key roles for these pathways in maintaining the integrity of the nervous system.

PPARα and PPARδ are regulators of fatty acid oxidation in a wide variety of tissues [124], [20]. PPARα was cloned as the molecular target of fibrates, cholesterol-lowering compounds that increase hepatic fatty acid oxidation [77]. The expression pattern of PPARα is restricted to tissues with a high capacity for fatty acid oxidation, such as heart, liver, brown adipose tissue (BAT), and oxidative muscle [16]. The function of PPARα seems to be more restricted to fatty acid uptake, beta-oxidation, and ketogenesis. In contrast, PPARδ is ubiquitously expressed, with higher levels in the digestive tract, heart, and BAT [52]. PPARδ plays a role in controlling oxidative metabolism and fuel preference, mainly in relation to fatty acid oxidation, mitochondrial OXPHOS, and glucose utilization [92], [98].

PPARγ is probably the most intensively studied of the three isoforms [160]. Knockout of PPARγ is embryonically lethal [143], [7]. PPARγ activation is associated with increased carbohydrate and lipid metabolism, reduction of plasma glucose concentration and stimulation of adipocyte differentiation [53]. PPARγ agonists are currently in clinical use for the management of type 2 diabetes [11], [168]. The physiological roles of PPARγ are highlighted in Table 1.

All PPARs can be activated by polyunsaturated fatty acids, but with different affinities [11], [44], [87]. Specific PPARγ ligands are divided into two major groups: [163], [26], [34]: (*i*) Endogenous or natural agonists and (ii) synthetic agonists. The endogenous agonists are divided into four subgroups: (i.1) The eicosanoids prostaglandin-A1 [8] and the cyclopentenone prostaglandin 15-deoxy-Δ^12,14^-Prostaglandin J2 (15D-PGJ2), which is the most naturally occurring and is commonly used as a potent ligand for PPARγ [180]; (i.2) unsaturated fatty acids such as Linoleic acid and Docosahexaenoic acid; (i.3) Nitroalkenes such as Nitrated oleic acid and Linoleic acid [140]; and (i.4). oxidized phospholipids such as hexadecyl azelaoyl phosphatidylcholine and Lysophosphatidic acid [39]. Non-steroidal anti-inflammatory drugs, as flufenamic acid, ibuprofen, fenoprofen, and indomethacin A, are also PPARγ ligands [11], [12], [178], [95].

(ii) The synthetic agonists are divided into five subgroups: (ii.1) The thiazolidinedione (TZD) family, which include Rosiglitazone, Pioglitazone and Troglitazone [88], [96]; (ii.2) the non-TZD agonists, such as Ciglitazone [122], Netoglitazone and Rivoglitazone; (ii.3) the dual α/γ agonists [2] also called glitazars (Include: Aleglitazar, Muraglitazar, Tesaglitazar and Saroglitazar); (ii.4) pan α/δ/γ agonists like bezafibrate; (ii.5) The selective PPARγ modulators (SPPARγM) such as metaglidasen/halofenate, PA-082, INT_131_ besylate and the angiotensin receptor antagonists [177]. The natural and synthetic ligands of PPARγ are listed in Table 1.

**Fig. 1:**
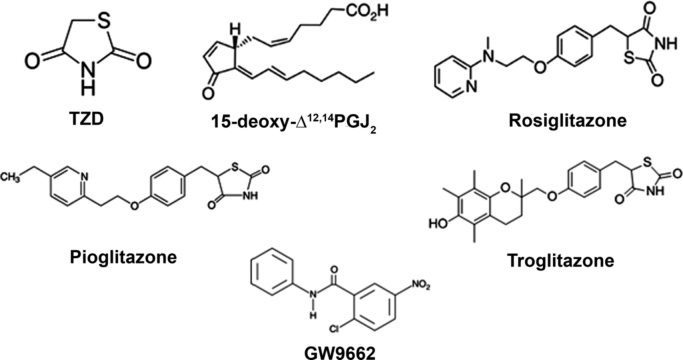
Chemical structures of TZD, 15-deoxy-Δ^12,14^-PGJ_2_, Rosiglitazone, Pioglitazone, Troglitazone and the antagonist GW9662.

The TZDs were the first ligand family discovered to bind and activate PPARγ with therapeutic benefit for diabetic patients [96]. However, the TZDs do have some adverse effects in man, including increased risk of heart failure, weight gain, increased cardiac load, peripheral oedema and haemodilution [136], [170]. While no endogenous antagonists for PPARγ have been reported, some synthetic antagonists are available, including GW9662, Bisphenol A Diglyceryl ether, LG100641, SR-202, and PD068235. The structure of some PPARγ agonist and antagonist are shown in Fig. 1.

## 2 Distribution of PPARγ

PPARγ is distributed in a number of tissues and cell types [133], [16], [43], demonstrated by in situ hybridization and by immunohistochemistry both in adult rat and in rat embryonic development [16], [17]. In man, PPARγ mRNA expression was abundant in adipose tissue, while lower level expression was seen in skeletal muscle [162]. PPARγ protein and mRNA were expressed at low levels in the adult rodent CNS, including neurons, astrocytes, microglia and oligodendrocytes [109], [12], [16], [38], [61]. High levels of expression of PPARγ have been found in discrete areas of the CNS, including the stellate cells of the cerebellar cortex, the olfactory tubercle, the piriform cortex, in rhomboid, centromedial, and parafascicular thalamic nuclei and in the reticular formation [109], [61]. PPARγ is highly expressed in dopaminergic cells in the basal ganglia [109], [38]. Other studies have demonstrated the presence of PPARγ protein by Western blot and immunohistochemistry in the midbrain [120], [22].

In cortical neurons, PPARγ immunoreactivity appears mainly as nuclear labelling, although cytoplasmic staining is sometimes detectable [109]. PPARγ is present at lower levels in liver, skeletal muscle and heart, but it is abundantly expressed in white adipose tissue. PPARγ is expressed in brown adipose tissue of rodents and in the CNS during foetal development [1], [107], [17]. The tissue expression of PPARγ is shown in Table 1.

## 3 PPARγ, adipose tissue, obesity and type 2 diabetes

The highest levels of PPARγ are expressed in adipose tissue [154], [25]. The expression and activation of PPARγ is sufficient to induce adipogenesis [155]. Adipose tissue can be divided into two major depots, white adipose tissue (WAT) and brown adipose tissue (BAT). In both WAT and BAT, PPARγ is a key regulator of differentiation. PPARγ plays an important role in regulating lipid metabolism in mature adipocytes. WAT stores excess energy as triglycerides, which can be mobilized by lipolysis to generate free fatty acids for use by other tissues, while BAT is the site of non-shivering thermogenesis, through activation of the brown adipocyte-specific protein, uncoupling protein 1 (UCP1). TZDs appear to co-ordinately activate gene expression leading to an increase in net lipid partitioning into adipocytes [157]. Target genes directly regulated by PPARγ, include lipoprotein lipase [139], fatty-acid transport protein [58], and oxidized LDL receptor 1 [31], favouring adipocyte uptake of phosphoenolpyruvate carboxykinase [156], [158], glycerol kinase [68], the glycerol transporter aquaporin 7 [73] and the circulating fatty acids.

Adipose tissue has generally been considered an organ used for storage and release of energy. Recent years have seen an explosive increase in the incidence of obesity worldwide, driving a resurgence of interest into the biology of adipose tissue, and of the adipocyte as regulators of body energy homoeostasis. PPARγ is a master regulator in the formation of fat cells and their normal function in the adult [134], [135]. PPARγ is induced during adipocyte differentiation, and forced expression of PPARγ in non-adipogenic cells effectively drives their differentiation into mature adipocytes [155], [157]. Adipose tissue accounts for about 10% of insulin-stimulated glucose disposal and has a key role in directing whole-body glucose homoeostasis. The discovery that the insulin-sensitizing TZDs are potent agonists for PPARγ [55], [96], suggested that apart from being a fatty acid sensor, PPARγ might serve an important role as a regulator of glucose metabolism. TZDs act by inhibiting hepatic glucose output and increasing insulin action to stimulate muscle glucose disposal in insulin-resistant animal models and patients [157].

Adipose tissue is dysfunctional in obesity, leading to progressive insulin resistance and risk for the development of type 2 diabetes. Phosphorylation of PPARγ at serine 273 by cyclin-dependent kinase 5 (Cdk5) stimulates diabetogenic gene expression in adipose tissues [30]. Inhibition of this modification is a key therapeutic mechanism of the TZDs. Insulin signalling activates the Akt/PI3K and Grb2/Ras/MEK/ERK kinase cascades. Recently it was demonstrated that ERK directly phosphorylates serine 273 of PPARγ and Cdk5 suppresses ERKs through direct action on a novel site in MEK, the ERK kinase. The pharmacological inhibition of MEK and ERK markedly improves insulin resistance in both obese and wild type ob/ob mice, and reversed the effects of the Cdk5 ablation, indicating that the ERK/Cdk5 axis controls the diabetogenic actions of PPARγ [5].

### 3.1 PPARγ coactivators

The binding of PPARγ to specific DNA sequences requires heterodimerization with a second member of the nuclear receptor family, the retinoic X receptor. Binding of agonist ligands to PPARγ triggers a conformational change that attracts transcriptional coactivators, which exist in multiprotein complexes and include members of the steroid receptor coactivator (SRC) family [103], [104]. SRC1 was originally identified as a coactivator of the steroid receptor superfamily [118]. SRC1 also interacts with PPARγ via LXXLL motifs in a ligand dependent manner [46]. The effects of SRC1 and SRC2 on metabolism are predominantly mediated by PPARγ coactivator 1α (PGC1α). PGC1α is upregulated in SRC2-depleted BAT. SRC1 and SRC2 control the energy balance between BAT and WAT. In BAT, SRC1 promotes energy expenditure via fatty acid oxidation [181]. SRC2 represses this process by activation of PPARγ in WAT, producing an increase in triglyceride accumulation in WAT and a decrease in free fatty acids.

PGC1α is the master regulator of mitochondrial biogenesis and plays a central role in coordinating and driving energy metabolism, fatty-acid oxidation, gluconeogenesis, glucose transport, glycogenolysis, peroxisomal remodelling, and oxidative phosphorylation [125], [126]. PGC1α also integrates and coordinates the activity of multiple transcription factors, such as nuclear respiratory factor 1 and 2 (NRF1–2), PPARα and mitochondrial transcription factor A (TFAM), which are all involved in mitochondrial biogenesis [127], [47].

PGC1α was originally identified in a two-hybrid screen using murine BAT cDNA and PPARγ as bait [127]. The interaction with PPARγ is ligand-independent and mediated through the N-terminal domain and an LXXLL motif [127], [97]. PGC1α also targets other transcription factors involved in metabolism, including FOXO1, SREBP, Foxa2 and Sox9 [126], [171], [85]. PGC1α works in concert with the oestrogen-related receptors (ERRα, ERRβ, ERRγ) which are members of the nuclear hormone receptor family which promote mitochondrial biogenesis in response to hormonal signals [47]. As well as promoting mitochondrial biogenesis, PGC1α also regulates the expression of several enzymes involved in antioxidant defence, detoxifying reactive oxygen species (ROS). These include superoxide dismutases 1 and 2 (SOD1–2), catalase and glutathione peroxidase-1 [146].

Other nuclear receptor coactivators are the histone acetyl transferases p300 and CREB-binding protein (CBP) [117], [6]. Both proteins interact with the ligand binding domain of PPARγ in a ligand- and LXXLL-dependent manner, while at the same time they interact with the transcriptional activation domain (AF-1) of PPARγ [64]. p300 and CBP enhance the transcriptional activity of both the AF-1 and AF-2 domains separately. CBP and p300 are also involved in adipogenesis.

PRDM16 is a BAT-specific transcription factor also involved in mitochondrial biogenesis [142]. It is a zinc finger protein which was first identified at a chromosomal breakpoint in myeloid leukaemia [116]. Overexpression of PRDM16 in cultured white preadipocytes resulted in activation of the brown adipose differentiation programme, the upregulation of mitochondrial genes, increase of mitochondrial biogenesis and uncoupled respiration [142]. Therefore, PRDM16 binds to PPARγ in a ligand-independent manner, works as a DNA binding transcription factor and enhances the transcriptional activity of PPARγ. PPARγ activation is required for the adipogenic function of PRDM16,.

## 4 Actions of PPARγ in rescuing mitochondrial function

Growing evidence suggests the involvement of mitochondrial dysfunction in the pathogenesis of many diseases, and especially in a number of major neurodegenerative and neuroinflammatory disorders. This dysfunction is associated with defects in mitophagy, in bioenergetics, in mitochondrial fusion/fission and mitochondrial trafficking, frequently also in combination with oxidative stress. Therefore, pharmacological pathways that promise to enhance mitochondrial function, reduce generation of reactive oxygen species (ROS) or enhance antioxidant defence represent important new therapeutic prospects for these diseases.

### 4.1 Beneficial actions of PPARγ on mitochondrial function

TZDs have been shown in many model systems to upregulate mitochondrial oxidative phosphorylation, mitochondrial biogenesis and antioxidant defence, serving together to protect cells from various forms of injury. Thus, TZDs were shown to protect lymphocytes from apoptosis following growth factor withdrawal, preventing the loss of mitochondrial membrane potential (ΔΨ_m_). The survival-enhancing effects were dependent on both the presence and activation of PPARγ [165]. During the development of obesity, in white adipocytes from ob/ob mice, which were treated with rosiglitazone showed mitochondrial remodelling, enhanced oxygen consumption and increased energy expenditure in white fat, suggesting that enhanced lipid utilization in this tissue may affect whole-body energy homoeostasis and insulin sensitivity [169]. In human adipocytes, both TZDs rosiglitazone and pioglitazone significantly increased mitochondrial DNA (mtDNA) copy number and expression of factors involved in mitochondrial biogenesis, including PGC1α and TFAM while also increasing the expression of genes in the fatty acid oxidation pathway, including carnitine palmitoyltransferase-1, malonyl-CoA decarboxylase, increased the expression of UCP-1 and medium-chain acyl-CoA dehydrogenase [14], [15]. A similar response was described in differentiated 3T3-L1 and C3H/10T1/2 adipocytes treated with rosiglitazone, which showed increased mitochondrial biogenesis, oxygen consumption and increased mitochondrial citrate synthase activity [132]. Additionally, pioglitazone stabilized MitoNEET, a protein that regulates oxidative capacity in 3T3-L1 cell line, rat brain mitochondria and COS-7 cells [119], [167], [33].

Protection by TZDs has particularly been explored in relation to the CNS, using a variety of model systems. Treatment with TZDs protected cortical astrocytes in culture against cell death induced by glucose deprivation, increasing glucose utilization, lactate production and mitochondrial membrane potential (ΔΨ_m_), demonstrating modification of astrocyte metabolism and mitochondrial function following PPARγ activation [42]. Pioglitazone and rosiglitazone prevented the death of differentiated SH-SY5Y cells (a neuroblastoma derived cell line), following glucose deprivation by promoting mitochondrial biogenesis, indicated by the up-regulation of PGC1α, NRF1, TFAM, cytochrome c oxidase subunit CO-I and CO-IV, and an increased mtDNA copy number, while there was no change in ΔΨ_m_ [108].

**Fig. 2:**
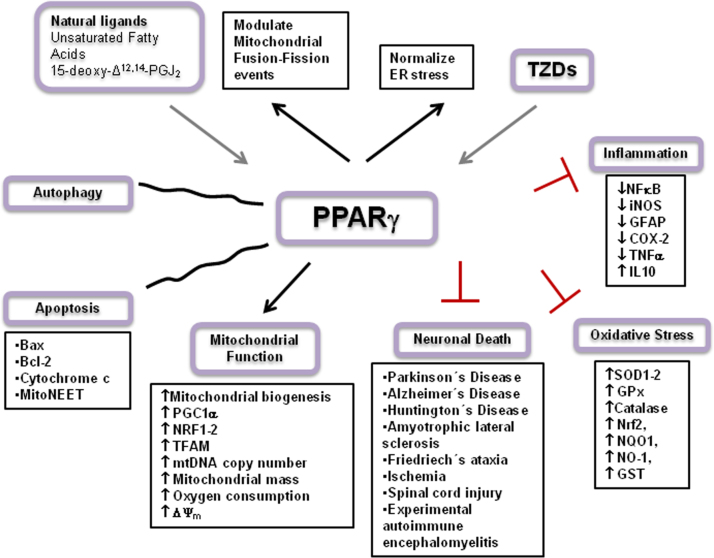
Activation of PPARγ increases mitochondrial biogenesis, oxygen consumption, ΔΨ_m_, antioxidant defences, regulates autophagy, and increases transcription factors, like PGC1α, NRF1–2, TFAM. Also PPARγ agonists regulate apoptosis and reduce inflammation. Thus, PPARγ agonists like TZDs regulate the expression of several target genes involved in neuronal survival and protected against neuronal death in several neurodegenerative disorders, mainly improving mitochondrial function and increasing redox capacity.

In the human neuron-like NT2 cell line, pioglitazone treatment also increased mtDNA content and the expression of nuclear-encoded electron transport chain subunit proteins. Oxygen consumption was increased as well as complex I and complex IV V(max) activities. Pioglitazone treatment was also associated with increased cytoplasmic, but reduced generation of mitochondrial, peroxide [65]. The natural ligand 15D-PGJ2 and rosiglitazone, both PPARγ ligands, increased glucose metabolism in rat cortical slices and astrocytes, restored brain ATP levels and prevented the impairment of glutamate uptake induced by repeated stress in rat brain [60]. In a more recent study, the PPARγ agonist ciglitazone protected the mitochondrial population of hippocampal neurons to oxidative stress (exposure to hydrogen peroxide). Protection was attributed to the modulation of mitochondrial fusion-fission events, suggesting that a PPARγ based therapy could have beneficial effects mediated by simultaneous actions on several complementary cellular pathways [182]. An schematic overview of PPARγ mechanisms is shown in Fig. 2.

### 4.2 Anti-oxidant and anti-apoptotic properties of PPARγ

Besides the protective effects of PPARγ on mitochondrial function, the protective effects of PPARγ activation have also been attributed to their anti-oxidant and anti-apoptotic properties. Thus, in hepatocytes, rosiglitazone enhanced the antioxidant defence of the cells, increasing the rate of elimination of ROS while increasing the expression of nuclear factor erythroid-derived 2-like 2 (Nrf2) and the antioxidant enzyme haem oxygenase-1 (HO-1) through the PPARγ-pathway [164].

Rosiglitazone was protective against neuronal damage by acetaldehyde, which causes oxidative neuronal death, inducing the generation of intracellular ROS and ultimately causing apoptotic cell death. The protection by rosiglitazone was attributed to the induction of the expression of anti-oxidant enzymes including SOD and catalase and by regulating the expression of B-cell lymphoma 2 (Bcl-2) and Bcl-2-associated X protein (Bax) [83]. In another study, treatment with pioglitazone was neuroprotective in rats treated with lipopolysaccharide (LPS), decreasing degeneration of dopaminergic pathways, decreasing inflammation and restoring mitochondrial function while also attenuating oxidative stress [74]. Rosiglitazone or PPARγ overexpression protected the neuronal N2A cell line against oxygen-glucose deprivation by maintaining ΔΨ_m_ and preventing apoptosis [172].

Rosiglitazone treatment also protected the neuroblastoma derived cell line, SH-SY5Y cells, against cytotoxicity induced by chlorpyrifos (an acetylcholinesterase inhibitor, widely used as pesticide). The protection was apparently mediated again by attenuation of oxidative stress and by anti-apoptotic effects, as well as the inhibition of the inflammatory cascade via inactivation of signalling by p38, nuclear factor-κB (NF-κB) and c-Jun N-terminal kinase [94].

Finally, in a model of bronchial asthma, oral treatment with rosiglitazone significantly increased anti-oxidant activity in lung tissue, associated with improved lung function, improved histopathological features and a significant decrease in the serum levels of IL-5 and IgE [51].

## 5 Mechanisms of protection mediated by PPARγ

Here, we will discuss the neuroprotective effects of PPARγ in relation to ischaemia, spinal cord injury, chronic neuropathic pain and experimental autoimmune encephalomyelitis (EAE), a model of Multiple sclerosis. We will also explore the literature relating to several neurodegenerative disorders including Amyotrophic lateral sclerosis (ALS), Huntington's disease (HD), Alzheimer’s disease (AD) and Parkinson's disease (PD), beyond their mechanisms on the mitochondrial function or on oxidative stress and apoptosis.

### 5.1 PPARγ actions on CNS ischaemic injury

PPARγ agonists may help to promote recovery after stroke. Endogenous neuronal PPARγ may be important in limiting damage after cerebral ischaemia, as PPARγ deficiency (PPARγ knock-out mice) was associated with increased brain damage and oxidative stress, a reduced expression of glutathione S-transferase, UCP-1, catalase and SOD1 after cerebral ischaemia [179]. In contrast, activation of PPARγ by the TZDs troglitazone or pioglitazone reduced inflammation and infarct volume and improved neurological function following middle cerebral artery occlusion in rats [149]. Treatment with rosiglitazone also reduced the infarct volume in a rat model of transient focal ischaemia [161]. Treatment with rosiglitazone significantly decreased DNA fragmentation, attenuated infarct volume, neurological deficits and neutrophilia after embolic focal cerebral ischaemia in rats [3]. Recently, 15D-PGJ2 a natural PPARγ ligand decreased neuronal autophagy by decreasing the expression of proteins LC3-II, Beclin 1, cathepsin-B, and LAMP1 after cerebral ischaemia reperfusion injury [174]. A single injection of rosiglitazone immediately following global cerebral ischaemia significantly increased neuronal survival and functional recovery while reducing the cerebral infarct volume and brain oedema [144]. All these data suggest that intrinsic PPARγ may be important in limiting stroke mediated injury and that further activation by TZDs may improve outcome.

### 5.2 PPARγ actions on pain and spinal cord injury

Neuropathic pain can be a major problem after spinal cord injury. Several studies suggest that TZDs can limit chronic severe pain and aid recovery from spinal injury. One study demonstrated that PPARγ is essential for coupling ibuprofen to RhoA inhibition and the subsequent promotion of neurite growth in primary neurons. Also in the same study, a similar mechanism accounted for the activation of PPARγ by ibuprofen in neuron-like PC12 and B104 cells, suggesting a molecular mechanism that could represent a novel therapeutic target in spinal cord injury [45]. Ajulemic acid, which directly interacts with PPARγ and has a potent analgesic and anti-inflammatory activity, may also be a potential candidate for drug development in the treatment of pain [4].

In animal models, rosiglitazone and pioglitazone, improved motor function, prevented myelin loss and reduced long term pain after spinal injury in adult rats [121]. In a mouse model of neuropathic pain, the intrathecal administration of rosiglitazone or the natural ligand 15D-PGJ2 reduced nerve injury-induced allodynia and hyperalgesia [32]. Additionally, pioglitazone reduced tactile allodynia and thermal hyperalgesia in mice subjected to peripheral nerve injury through attenuation of proinflammatory cytokine secretion [100]. Intracerebroventricular administration of rosiglitazone or 15D-PGJ2 reduced peripheral inflammation in the brain, reduced inflammatory pain, induced the expression of Fos in the dorsal horn, and inhibited local oedema [110].

Treatment of animals subjected to spinal cord injury with rosiglitazone significantly increased the proliferation of neuronal precursor cells, accelerated locomotor recovery and reduced NF-κB expression [105]. Local administration of rosiglitazone significantly reduced hypersensitivity to heat and mechanical stimuli, and paw swelling and reduced the development of post-incisional pain, possibly by regulating macrophage polarity at the inflamed site [71]. Attenuation of the development of inflammatory pain by rosiglitazone was attributed to the induction of haem oxygenase 1 (HO-1) in macrophages, and gene induction of endogenous opioid proenkephalin [70]. In rats with neuropathic pain, oral or intraperitoneal administration of pioglitazone limited multiple behavioural signs of somatosensory hypersensitivity and significantly reduced touch stimulus-evoked phospho-extracellular signal-related kinase (*p*-ERK) [111].

### 5.3 PPARγ actions on EAE

Experimental allergic encephalomyelitis is often used as a model of demyelinating disease. The pathophysiology of the disease model is complex, but a significant body of data suggests key roles of mitochondrial dysfunction and oxidative and nitrosative stress [166], [56], [57]. In two models of EAE (immunized with myelin oligodendrocyte glycoprotein peptide and with myelin basic protein), treatment with pioglitazone delayed neurodegeneration by decreasing lymphocyte infiltration, reduced demyelination, chemokine and cytokine expression and increased inhibitor of kappa B expression in the brain [54].

## 6 Mechanisms of protection by PPARγ in neurodegenerative diseases

### 6.1 Impact of PPARγ in Amyotrophic lateral sclerosis

Amyotrophic lateral sclerosis (ALS, also known as motoneuron disease, MND), is a neurodegenerative disease that causes paralysis and shortened lifespan due to the selective death of motoneurons. Extensive data point to a major role for calcium mediated mitochondrial toxicity and oxidative stress in ALS including a major role for excitotoxicity, which is a prime example of calcium dependent mitochondrial toxicity [36], [37], [78]. The effects of the PPARγ agonist troglitazone have been explored in rat motoneurons, which showed improved survival. However, troglitazone did not promote the survival of sensory, sympathetic, septal or hippocampal neurons [115]. These results indicate specific neurotrophic activity of troglitazone for motoneurons through the activation of phosphatidylinositol 3-kinase (PI3-kinase) and supported the potential value of troglitazone for the treatment of motoneuron diseases such as ALS.

Mutations of SOD1 underlie MND in a rare subgroup of patients with familial disease. The specific mechanism of MND/ALS caused by mutations of SOD1 remain unclear – they are not simply due to loss of antioxidant function but seem to involve a toxic gain of function [137], [176]. Pioglitazone protected transgenic mice carrying the ALS related SOD1-G93A mutation from motoneuron neurodegeneration, improving muscle strength and body weight and delaying disease onset [141]. In SOD1-G93A transgenic mice, treatment with pioglitazone extended survival, reduced gliosis and also reduced iNOS, NF-κB, and 3-nitrotyrosine immunoreactivity in the spinal cord, suggesting a significant role against nitrosative stress [86]. In another study of the same mouse model, pioglitazone also protected motoneurons against p38-mediated neuronal death and NF-κB-mediated glial inflammation [145]. The accumulation of critical concentrations of lipid peroxidation adducts during the progression of ALS leads to the activation of PPARγ in motoneurons of SOD1-G93A mice. This in turn triggers self-protective mechanisms that involve the up-regulation of lipid detoxification enzymes, including lipoprotein lipase and glutathione S-transferase α-2 [9].

Recently, in a Drosophila model of ALS based on TAR DNA-binding protein 43 (TDP-43) that recapitulates several aspects of ALS pathophysiology, pioglitazone rescued TDP-43-dependent locomotor dysfunction in motoneurons and glia but not in muscles and also was neuroprotective when FUS, but not SOD1, is expressed in motoneurons [81].

Finally, a randomized, double blind, placebo-controlled trial of pioglitazone in combination with riluzole in ALS patients, disappointingly found that treatment with pioglitazone had no positive benefit, with no increase in the survival of ALS patients treated with riluzole [50].

### 6.2 Impact of PPARγ in Huntington's disease

Huntington's disease (HD) is an autosomal dominant progressive neurodegenerative disorder associated with progressive extrapyramidal motor dysfunction. It is a genetic disease, caused by mutations that cause CAG trinucleotide expansion in the exon-1 region of the huntingtin gene. Extensive data links huntingtin mutations and CAG repeats to mitochondrial damage and oxidative stress [138], [66], [69].

The protective effects of PPARγ agonists have been studied in several different models of HD. In striatal cells in culture expressing mutant huntingtin STHdh(Q111), rosiglitazone increased mitochondrial biogenesis and protected against calcium dependent (thapsigargin-induced) mitochondrial dysfunction [128]. Similarly, in cultured rat primary cortical neurons expressing mutant huntingtin STHdh(Q111), PPARγ activation by rosiglitazone significantly attenuated thapsigargin-induced cell death, concomitant with reduced caspase activation, a delay in the loss of ΔΨ_m_, and a reduction of ROS generation [80]. In addition, a reduction in mitochondrial mass associated with the expression of mutant huntingtin in N2A cells, was rescued by rosiglitazone [29]. Rosiglitazone also significantly increased survival in N2A cells expressing mutant huntingtin, while also upregulating the expression of PPARγ, PGC1α, NRF-1, TFAM and improving mitochondrial function in mutant cells. Rosiglitazone treatment also normalized endoplasmic reticulum stress sensors Bip, CHOP and ASK1 and significantly reduced mutant huntingtin aggregates that included ubiquitin and heat shock factor 1 and increased the levels of the functional ubiquitin-proteasome system and heat shock protein 27/70 [28].

PPARγ activation by pioglitazone protected striatal cells from mitochondrial dysfunction and oxidative stress in a 3 nitropropionic acid (3-NP)-induced experimental model of HD (3-NP, irreversibly inhibits succinate dehydrogenase and produces in animals behavioural, biochemical and morphologic changes considered to be similar to those occurring in HD). The beneficial effects on mitochondrial dysfunction were attributed to interference with the NF-κB signalling pathway, which has been implicated in the pathogenesis of HD [112].

In a mouse model R6/2 of HD, treatment with rosiglitazone rescued progressive weight loss, motor deterioration, formation of mutant huntingtin aggregates, and reduced the expression of two neuroprotective proteins, the brain derived neurotrophic factor (BDNF) and Bcl-2 [27]. Bezafibrate, a pan-PPAR agonist, increased mitochondrial biogenesis, increased the numbers of muscle mitochondria, improved survival, improved the phenotype and reduced brain, muscle and BAT pathology in transgenic mouse R6/2 [82]. Treatment with rosiglitazone also reduced huntingtin aggregates and normalized the expression of PGC1α, NRF1–2, and TFAM in the cortex of a transgenic mouse R6/2 [29]. Moreover, chronic administration of rosiglitazone to N171–82Q HD mice significantly improved motor function, attenuated hyperglycaemia, rescued BDNF deficiency in the cerebral cortex, prevented loss of orexin-A-immunopositive neurons in the hypothalamus, prevented PGC1α reduction and increased Sirt6 protein levels [79].

### 6.3 Potential benefits of PPARγ activators in Alzheimer’s disease

Alzheimer’s disease (AD) is a neurodegenerative disorder characterized by a progressive cognitive decline, associated with intracerebral aggregates of amyloid-β (Aβ) protein and the accumulation of the hyperphosphorylated tau protein. Substantial data point to oxidative stress and mitochondrial dysfunction as major contributors to the pathophysiology, although there is significant controversy about the relative roles of potential underlying mechanisms [21], [49].

Acute treatment of APPV717I transgenic mice model of AD with pioglitazone or ibuprofen significantly decreased the number of activated microglia and astrocytes in the hippocampus and cortex. Both drugs reduced the expression of the proinflammatory enzymes COX-2 and iNOS and reduced Aβ deposits in the hippocampus and cortex [72]. In APPV717I transgenic mice, pioglitazone treatment significantly attenuated astroglial activation, reduced signs of oxidative stress and normalized the cerebral blood flow and glucose uptake responses to increased neuronal activity, but failed to improve spatial memory [114]. In AD Apolipoprotein E (ApoE) knockout mice, rosiglitazone increased mitochondrial biogenesis as demonstrated by the induction of both oestrogen-stimulated related receptor alpha (ESRRA) mRNA and mtDNA copy number and improved glucose utilization [147]. Treatment with rosiglitazone improved cognition in the Tg2576 mice, another transgenic mouse model of AD which presents accumulation of Aβ, neuronal loss, and cognitive decline [131]. In the Tg2576 mice, rosiglitazone enhanced cognitive function and normalized dentate granule cell presynaptic function, through regulation of presynaptic vesicular proteins critical for glutamatergic neurotransmitter release, synaptic transmission, and short-term plasticity [113].

In another mouse model of AD, the APPswe/PS1Δe9 mice, treatment with pioglitazone reduced brain levels of soluble and insoluble Aβ levels which correlated with the loss of both diffuse and dense-core plaques within the cortex [101]. In another study using the same APPswe/PS1Δe9 mice, the novel selective PPARγ modulator, DSP-8658 and pioglitazone, improved spatial memory performance and increased microglial Aβ phagocytosis which subsequently resulted in a reduction of cortical and hippocampal Aβ levels. The phagocytosis of Aβ was mediated through the upregulation of scavenger receptor CD36 [175]. Activation of Wnt signalling by lithium and rosiglitazone reduced spatial memory impairment and neurodegeneration in brains of APPswe/PS1Δe9 mice, and both drugs also prevented changes in presynaptic and postsynaptic marker proteins [153].

In Cdk5 conditional knock-out mice, astrogliosis, microgliosis, neuronal loss and behavioural deficit were all improved by rositglitazone. Cdk5 plays an important role in neurogenesis and there is some evidence that it participates in the pathogenesis of AD [159].

PPARγ activation by troglitazone and rosiglitazone protected rat hippocampal neurons in culture against toxicity through exposure to synthetic Aβ_1–40_ peptide (5 μM), resulting in the modulation of Wnt signalling components, including the increase of the cytoplasmic and nuclear β-catenin levels and the inhibition of glycogen synthase kinase-3β (GSK-3β), suggesting that GSK-3β is responsible for the tau hyperphosphorylation in AD [75]. Furthermore, in cultured hippocampal neurons, rosiglitazone was protective against mitochondrial damage, oxidative stress and apoptosis induced by 5 μM of synthetic Aβ_1–40_ peptide, through a mechanism involving elevated expression of Bcl-2. Also, PPARγ overexpression prevented H_2_O_2_-induced ROS, stabilized mitochondria, and decreased Aβ-induced toxicity [59].

In a clinical trial, patients with mild-to-moderate AD were randomized to placebo or rosiglitazone treatment and the exploratory analyses suggested an improvement in cognitive and functional measures in ApoE ε4 non-carriers, whereas no improvement and some decline was noted in the ApoE ε4 allele carriers [130]. In a further randomized, double-blind, placebo-controlled phase III study in mild-to-moderate AD, rosiglitazone monotherapy failed to show any benefit in cognition or in global function in ApoE ε4 negative subjects [67].

In addition, in a randomized pilot clinical trial of the safety of pioglitazone in non-diabetic patients with AD, pioglitazone was generally well tolerated with an increase in peripheral oedema (oedema is a commonly recognized effect of pioglitazone in clinical use and this potential adverse treatment effect was anticipated) and there were no other serious or unanticipated adverse events or clinical laboratory changes attributable to pioglitazone over a long-term exposure [63]. Some clinical trials of different PPARγ agonist for the treatment of neurodegenerative diseases are shown in Table 2.

### 6.4 Impact of PPARγ in Parkinson's disease

Parkinson's disease (PD) is characterized by the progressive neurodegeneration of dopaminergic neurons of the substantia nigra, causing a characteristic tremor, bradykinesia and a slower deterioration of cognitive function. There are extensive data pointing to impaired mitochondrial function and oxidative stress as key mechanisms in the pathophysiology of the disease, most notably pointing to complex I damage, impaired autophagy and calcium dependent mitochondrial free radical generation for reviews see: [148], [49], [99]. The neuroprotection of PPARγ activation has been studied in a number of models of PD.

In human neuroblastoma cells in culture, rosiglitazone was protective against mitochondrial dysfunction produced by the complex I inhibitor 1-methyl-4-phenylpyridinium ion (MPP^+^), increasing ΔΨ_m_, while also increasing the expression of SOD and catalase, and the expression of Bcl-2 and Bax, thus promoting both antioxidant defence and limiting apoptosis [84]. 15D-PGJ2 and pioglitazone preserved mitochondrial function of oligodendrocyte progenitors against the inhibitory activities of rotenone and cytokine tumour necrosis factor α (TNFα), by maintaining ΔΨ_m_, reducing mitochondrial ROS production and by increasing the expression of PGC1α, uncoupling protein 2 (UCP-2) and cytochrome oxidase subunit COX1. TNFα decreased Ca^2+^ oscillations, as well as the frequency of the oscillatory events and the TNFα effects were prevented by pioglitazone, also pioglitazone protected the oligodendrocyte progenitor differentiation to immature oligodendrocyte [40]. In differentiated neuroblastoma SH-SY5Y cells associated with chronic partial inhibition of complex I with rotenone and with loss of PINK1 function, as cellular models related to sporadic and familial PD respectively, rosiglitazone increased mitochondrial biogenesis, increased oxygen consumption, increased mitochondrial mass, ΔΨ_m_, mtDNA copy number, decreased autophagy and suppressed free radical generation [35]. A novel thiobarbituric-like compound MDG548, which acts as a functional PPARγ agonist, was tested in vitro and in vivo models of PD, MDG548 protected against H_2_O_2_ and MPP^+^ neurotoxicity in PC12 cells and in the mice treated with MPTP, the MDG548 reduced reactive microglia and iNOS induction in the substantia nigra [91].

1-methyl-4-phenyl-1,2,3,6-tetrahydropyridine (MPTP) administration is used in animals to model PD, as it causes selective death of dopaminergic neurons. The oxidative stress produced by this toxin was reduced by pioglitazone treatment as evidenced by a reduction in the levels of malondialdehyde and increased glutathione levels [89]. In response to MPTP plus probenecid, treatment with rosiglitazone was effective in protection against partial degeneration of the substantia nigra and the decline of striatal dopamine. Also, the production of the pro-inflammatory cytokine TNFα by microglia was inhibited by rosiglitazone [22]. In another study, rhesus monkeys were subjected to an intracarotid injection of MPTP and pioglitazone was also shown to be neuroprotective when administered early. Behavioural recovery was associated with preservation of dopaminergic markers and reduced infiltration by CD68-positive macrophages in the nigrostriatal area and by a significant improvement in a clinical rating score [152].

A novel non-TZD partial PPARγ agonist LSN862, was shown to be neuroprotective in MPTP-induced neurodegeneration, which was associated with the modulation of PPARγ and PGC1α expression, the downregulation of neuroinflammation and decreased oxidative stress [151]. The expression of PPARγ in the dopaminergic neurons of the substantia nigra pars compacta and putamen of hemiparkinsonian nonhuman primates was prominent and the dopaminergic neurons were more likely to survive against MPTP-induced damage after activation by pioglitazone [150]. In the MPTP-probenecid mouse model, the PPARγ agonists may act through an immunomodulatory action, since the anti-inflammatory microglia were stimulated and inflammatory microglia were inhibited in the substantia nigra compacta by rosiglitazone [123]. Pioglitazone protected against deterioration of motor function and neuronal loss in the acute MPTP mouse model, but failed to protect in a rat model using 6-hydroxydopamine (6-OHDA) as a dopaminergic selective toxin, thought to reflect the severity of damage caused by 6-OHDA [90]. The activation of PPARγ receptors by rosiglitazone in the 6-OHDA-lesioned rat, significantly increased GFAP expression in the striatum and attenuated COX-2 and TNFα expression [93].

In 2013, pioglitazone entered in a multicenter placebo-controlled phase II study, in early PD (NCT01280123) and depending on the study results, a subsequent phase III trial could follow. Recently, in a retrospective cohort study, the prescription of the PPARγ agonist glitazone in patients with diabetes was associated with a reduction in the incidence of PD, suggesting that PPARγ pathways may be a successful drug target in PD [18].

### 6.5 Impact of PPARγ in Friedreich’s ataxia

The effect of PPARγ activation has also been studied in Friedreich’s ataxia, an autosomal recessive disorder caused by mutations of the protein frataxin. The disease phenotype includes a progressive cardiomyopathy which leads eventually to heart failure. It was demonstrated that PPARγ agonist increased both messenger RNA and protein levels of frataxin [102]. Pioglitazone, which seems to have fewer side effects than rosiglitazone is on a phase III clinical trial for its neuroprotective properties. The project, titled "Effect of pioglitazone administered to patients with Friedreich’s ataxia: proof of concept". However, the antecedents and possible consequences should be taken into consideration when PPARγ agonist drugs are considered as possible therapeutic agents for FRDA patients [62].

## 7 Conclusion

The modulation of molecular pathways that include mitochondrial dysfunction, oxidative stress, process of autophagy, inflammation and apoptosis, which are implicated in the pathophysiology of many major diseases including neurodegenerative disorders seem to present very valuable therapeutic targets. In a similar vein, PPARγ agonists have been showed to have a wide range of activities that positively influence the pathology of diseases in several experimental models, and have the capacity to be neuroprotective by regulating the expression of genes involved in neuronal survival processes and therefore promote the health of neurons. PPARγ agonists could be valuable potential therapeutic targets for several diseases including neurodegenerative disorders, since the results from patients and from in vivo and in vitro models of neurodegenerative disorders underline the beneficial effects of PPARγ agonists for future clinical trials. However, further studies are needed if we are to understand the potential of PPARγ agonists for patient benefit or the mechanisms by which PPARγ exerts its protective actions.

## Figures and Tables

**Table 1:** Natural and synthetic ligands, physiological role, and tissue expression of PPARγ.

Natural ligands	Refs.	Synthetic ligands	Refs.	Tissue expression	Refs.	Physiological role	Refs.
Unsaturated Fatty Acids	[44]	Pioglitazone	[88]	High-Adipose tissue (white and brown)	[1], [155]	Glucose homoeostasis	[133], [42]
Prostaglandin A1	[8]	Rosiglitazone	[96]	Low-Intestines, Liver, Retina	[16]	Lipid storage	[155]
15-deoxy-Δ^12,14^-PGJ_2_	[180]	Troglitazone	[96]	Low-Heart	[16]	Differentiation and maturation of adipocytes	[155]
Nitroalkenes	[140]	Ciglitazone	[122]	Low-Muscle	[162]	Fatty acid oxidation	[14], [15]
Oxidized phospholipids	[39]	Glitazars	[2]	Low-Brain (oligodendrocytes, neurons, astrocytes, microglia)	[109], [61]		
		PPARγ modulators	[177]				
		Non-steroidal anti-inflammatory drugs	[95]				

**Table 2:** Some clinical trials of TZDs for the treatment of different neurodegenerative diseases.

**Agent**	**Disease**	**Outcome**	**Refs.**
Rosiglitazone+Riluzole	ALS	Had no positive benefit	[50]
Rosiglitazone	AD	No improvement in ApoE ε4 allele carriers	[130]
Rosiglitazone	AD	No benefit in cognition or in global function in ApoE ε4 negative subjects	[67]
Pioglitazone	AD	Well tolerated but an increase in peripheral oedema	[63]
Glitazone	PD	A reduction in the incidence of PD in patients with diabetes	[18]
Pioglitazone	PD	Is unlikely to modify progression in early PD	[76]
